# Numerical Investigation of DC Dielectrophoretic Deformable Particle–Particle Interactions and Assembly

**DOI:** 10.3390/mi9060260

**Published:** 2018-05-25

**Authors:** Xiang Ji, Li Xu, Teng Zhou, Liuyong Shi, Yongbo Deng, Jie Li

**Affiliations:** 1 Mechanical and Electrical Engineering College, Hainan University, Haikou 570228, China; jixiangsci@outlook.com; 2 School of Energy and Power Engineering, Wuhan University of Technology, Wuhan 430070, China; jieli@whut.edu.cn; 3 State Key Laboratory of Applied Optics, Changchun Institute of Optics, Fine Mechanics and Physics (CIOMP), Chinese Academy of Sciences, Changchun 130033, China

**Keywords:** dielectrophoresis, particle assembly, particle interactions, arbitrary Lagrangian–Eulerian (ALE), microfluidics, fluid–structure interaction, Navies–Stokes equation

## Abstract

In a non-uniform electric field, the surface charge of the deformable particle is polarized, resulting in the dielectrophoretic force acting on the surface of the particle, which causes the electrophoresis. Due to dielectrophoretic force, the two deformable particles approach each other, and distort the flow field between them, which cause the hydrodynamic force correspondingly. The dielectrophoresis (DEP) force and the hydrodynamic force together form the net force acting on the particles. In this paper, based on a thin electric double layer (EDL) assumption, we developed a mathematical model under the arbitrary Lagrangian–Eulerian (ALE) numerical approach method to simulate the flow field, electric field, and deformable particles simultaneously. Simulation results show that, when two deformable particles’ distances are in a certain range, no matter the initial position of the two particles immersed in the fluid field, the particles will eventually form a particle–particle chain parallel to the direction of the electric field. In actual experiments, the biological cells used are deformable. Compared with the previous study on the DEP motion of the rigid particles, the research conclusion of this paper provides a more rigorous reference for the design of microfluidics.

## 1. Introduction

Dielectrophoresis (DEP) refers to the phenomenon of force acting on the low permittivity dielectric particles under a spatially non-uniform electric field [1,2,3,4,5,6]. The non-uniform electric field polarizes the dielectric particles, and produces the DEP force on the particle surface, which moves particles in the fluid medium. The magnitude of the DEP force is not related to whether the particles are charged or not [7,8,9,10]. The DEP force is highly related to the size and electrical properties of the particles, the electrical properties of the fluid medium, and the parameters of the applied electric field. DEP assembly of colloidal particles has become one of the important technologies for micro-nano scale particles manipulation in microfluidics [11,12,13,14,15,16,17,18,19].

For the precision of DEP particle manipulation, the particle–particle interaction should not be ignored [20,21,22,23,24,25,26,27]. A pair of particles is formed when, for example, the presence of one particle distorts the local electric field, resulting in a non-uniform electric field around another particle. This non-uniform electric field polarizes another particle and produces a DEP force, which is known as particle–particle interaction DEP force. The interaction DEP force drives the two particles approach each other and eventually form a particle–particle chain parallel to the applied electric field, which is the basis of DEP assembly technology.

Experimental study on the DEP particle–particle interaction was once performed. Two spherical particles initially presenting a certain angle with respect to the applied electric field, after a period of DEP attractive motion, eventually formed a particle–particle chain parallel to the applied electric field. Additionally, the DEP interactive motion of two rod-shaped particles was simulated as well. Simulation of the two spherical particles’ DEP motion, where the gap between the two particles was larger than the particle radius, was studied by Kang and Li [23]. In their work, the velocity, trajectories, and force of the two particles were analyzed simultaneously. Qian once studied the particle–particle interactions and their motions numerically [23,24]. The numerical simulation results show that negative DEP motion, from higher to lower regions, always tends to attract particles and finally forms a particle–particle chain parallel to the applied electric field.

However, in the above numerical studies, the particles were considered rigid objects, but in the actual experimental studies, the biological particles used are usually deformable [10,18,28,29,30,31]. Studies on the DEP motion of deformable particles have therefore become highly important. In this paper, the DEP motion of a pair of deformable particles under a direct current (DC) electric field is simulated. The motion of different elastic modulus particles is compared and the velocity, trajectory, and force of particle–particle DEP motion are analyzed in detail. We developed a multiphysics model based on the thin electric double layer (EDL) assumption. The fluid flow field, the DC electric field, and the motion of deformable particles are solved simultaneously with an arbitrary Lagrangian–Eulerian (ALE) numerical method [11,18,32,33]. Meanwhile, the DEP force is calculated using the Maxwell stress tensor method, which has been demonstrated as the most rigorous approach to evaluate the DEP force.

## 2. Materials and Methods

### 2.1. Mathematical Model

**Figure 1:**
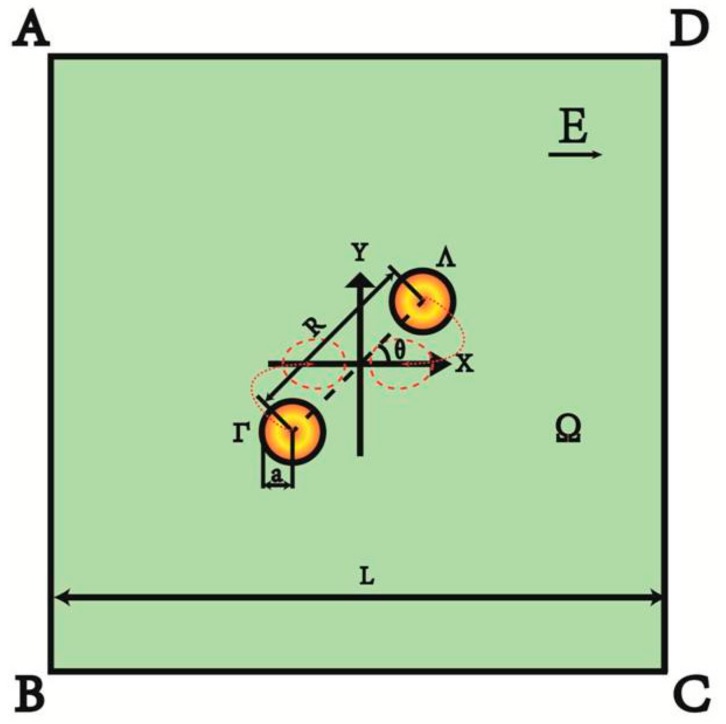
Two identical particles suspended in a square domain Ω under an externally applied electric field **E**. The origin of the 2D Cartesian coordinate systems (*x*, *y*) is located at the midpoint of the connecting line of the two particles and the center of the square. The particle radius and the distance between the two particles are denoted by A and R, respectively. The angle between the connecting line of the two particles and the *x*-axis is denoted by *θ*. The surfaces of the particles in the first quadrant and the third quadrant are denoted by Λ and Γ, respectively. The dashed lines show the locations and shapes of the two particles. The dotted lines show the trajectories of the interaction motion.

To develop a mathematical model, we consider two identical circular particles suspended in an incompressible Newtonian fluid confined in a square domain Ω with density *ρ* and dynamic viscosity *μ*, as shown in Figure 1. The midpoint of the two particles’ connecting line coincides with the center of the square domain, which is selected as the origin of the 2D Cartesian coordinate system (*x*, *y*). The side length of the square and the radius of the particles are, respectively, *L* and *a*. The center-to-center distance of the two particles and the angle between the *x*-axis and the connecting line of the two particles are, respectively, *R* and *θ*. The surface of the two particles suspended in the first quadrant and the third quadrant are, respectively, Γ and Λ. The domain enclosed by the sides of the square except for the particle is selected as the computational domain Ω. An electric field, *E*, generated by an externally electric potential applied from inlet AB to outlet CD, induces the dielectrophoretic particle–particle interactions and their relative motion of the particles. The dashed lines show the location and shape of the two particles under one time step during their DEP motion, and the dotted lines show the trajectories of their motion.

Considering that the electric double layer (EDL) thicknesses adjacent to the particle surface and the channel wall are very thin in comparison to the particle radius and the distance between AD and BC, the thin EDL approximation is applied. Therefore, the net charge density in computational domain Ω is zero. The electrical potential ϕ in the computational domain can be described by the Laplace equation, given as

(1) $$\nabla^{2}\phi =0\;\mathrm{in}\;\Omega \;.$$

The electric filed **E** is given as

(2) E=−∇ϕ in Ω .

Electric potential applied to generate the electric field **E** is given as

(3) $$\phi =\phi_{0}\;\mathrm{on}\;\mathrm{AB}\;,$$

and

(4) ϕ=0 on CD .

Electric insulation applied on all the other boundaries, including the particle surface Γ and Λ, is given by

(5) n⋅∇ϕ=0 on AD, BC, Γ and Λ ,

where **n** is the unit outward normal vector on the corresponding boundary.

The Reynolds number of the fluid flow in this study is very small; as a result, the inertia terms in the Navier–Stokes equations are neglected. The motion of the fluid is governed by the continuity equation and the Stokes equations, given as

(6) $$\nabla \cdot u=0\;\mathrm{in}\;\Omega_{f},$$

and

(7) $$\rho \frac{\partial u}{\partial t}=\nabla \cdot [-pΙ+\mu (\nabla u+\nabla u^{T})]\;\mathrm{in}\;\Omega_{f}\;,$$

where **u** is the fluid velocity vector, **I** is the unit tensor, and $\nabla u^{T}$ is the transpose of the velocity gradient ∇u. The quantities *ρ*, *μ*, *p* represent, respectively, the fluid density, dynamic viscosity, and the pressure.

An open boundary condition is applied on the two opening boundaries AB and CD, which are selected as inlet and outlet of the computational domain Ω, given as

(8) $$\nabla \cdot \left[-pI+\mu \left(\nabla u+\nabla u^{T}\right)\right]=0\;\mathrm{on}\;\mathrm{AB}\;\mathrm{and}\;\mathrm{CD}\;.$$

An symmetry boundary condition is applied on the other boundaries AD and BC, given as

(9) $$k=[\mu \left(\nabla u+{\left(\nabla u\right)}^{T}\right)]\;n\;\mathrm{on}\;\mathrm{AD}\;\mathrm{and}\;\mathrm{BC},$$

where the quantity **k** is k−(k⋅n)n=0,u⋅n=0.

The velocity on the surface of one of the two particles is denoted by $u_{pi}$ and consists of two parts, that is, the Smoluchowski slip velocity, which arises from the surface charge of the particle, and the velocity related to the motion of the particle. The quantity $u_{pi}$ is given as

(10) $$u_{pi}=\frac{\varepsilon_{f}\zeta_{pi}}{\mu }\left(I-nn\right)\cdot \nabla \phi +\frac{\partial S}{\partial t}\;\mathrm{on}\;\Gamma \;\mathrm{and}\;\Lambda ,$$

where the quantities $\zeta_{pi}$ and **S** are, respectively, the zeta potential and the displacement of the particle, and the quantity **S** can be calculated by

(11) $$\rho_{p}\frac{\partial^{2}S}{\partial t^{2}}-\nabla \cdot \sigma \left(S\right)=0\;\mathrm{in}\;\Omega_{p}\;,$$

where $\rho_{p}$ is the density of the particle, and the quantity σ(S) represents the Cauchy stress of the solid phase, which is a function of the displacement of the particles.

Considering that the particles are incompressible Neo-Hookean material, which can be described using the strain energy density function, given as

(12) $$W=\frac{G_{0}}{2}\left(I_{C}-3\right)\;,$$

where *G*_0_ is the shear modulus of the deformable particles, $I_{C}=tr\left(C\right)$ is the first invariant of the right Cauchy-Green tensor, where $C=F^{T}F$, and the quantity **F** is the deformation gradient tensor, which can be calculated by F=∇S+I.

The corresponding Cauchy stress of the Neo-Hookean material can be described by

(13) $$\sigma \left(S\right)=J^{-1}PF^{T}\;,$$

where **J** is the determinant of the deformation gradient tensor **F**, J=1 for an incompressible Neo-Hookean material, and $P=\partial W_{s}/\partial \nabla_{X}S$ is the first Piola–Kirchhoff stress.

The traction force on the particle–fluid interface, which consists of hydrodynamic and electrokinetic stresses is continuous, and can be described by

(14) $$\sigma_{p}\cdot n_{p}=\sigma_{f}\cdot n_{f}+\sigma_{E}\cdot n_{f}\;,$$

where $\sigma_{p}$ and $n_{p}$ are, respectively, total stress tensor on the particle surface and the unit normal vector directed from the particle surface into the fluid. The quantities $\sigma_{f}=-pI+\mu \left(\nabla u+\nabla u^{T}\right)$ and $\sigma_{E}=\varepsilon_{f}EE-\frac{1}{2}\varepsilon_{f}\left(E\cdot E\right)I$ represent, respectively, the hydrodynamic stress tensor and the Maxwell stress tensor.

The quantities in the governing equations can be normalized by characteristic length scale, characteristic electric potential, and characteristic velocity, which can be denoted as particle radius *a*, electric potential $\phi_{0}$, and particle velocity $U_{\infty }=\frac{\varepsilon_{f}\phi_{0}}{\mu }\frac{\phi_{0}}{a}$, respectively. Therefore, the dimensionless governing equations and the boundary condition are expressed as follows:

(15) $${\nabla^{*}}^{2}\phi^{*}=0\;\mathrm{in}\;\Omega_{f}$$

(16) $$\phi =\frac{\phi }{\phi_{0}}\;\mathrm{on}\;\mathrm{AB}$$

(17) ϕ=0 on CD

(18) $$n\cdot \nabla^{*}\phi^{*}=0\;\mathrm{on}\;\mathrm{AD},\;\mathrm{BC},\;\Gamma \;\mathrm{and}\;\Lambda$$

(19) $$\nabla^{*}\cdot u^{*}=0\;\mathrm{in}\;\Omega_{f}$$

(20) Re∂u∗∂t∗=−∇∗p∗+∇∗2u∗ in Ωf

### 2.2. Numerical Method and Code Validation

To ensure the accuracy and feasibility of the simulation results, some important parameters of the particle, flow field, and electric field used in this study are listed. Since the effect of particle gravity is not considered in this paper, the particle density is set to be the same as the density of the fluid, $\rho_{p}=1.0\times 10^{3}{\;\mathrm{kg}/m}^{3}$. The radius of the particle is *a* = 10 μm. The particle permittivity and conductivity are set as $\varepsilon_{p}=2.6\varepsilon_{0}$ and $\sigma_{p}=4.0\times 10^{-4}\;S/m$, respectively, where the quantity $\varepsilon_{0}$ is the permittivity in vacuum [23]. The fluid medium used in this simulation is water. Its density and dynamic viscosity are set as $\rho_{f}=1.0\times 10^{3}\mathrm{kg}/m^{3}$ and $\eta =1.0\times 10^{-3}\;\mathrm{kg}/\left(m\cdot s\right)$, respectively. Besides, the fluid permittivity and conductivity are set as $\varepsilon_{f}=80\varepsilon_{0}$ and $\sigma_{f}=2.0\times 10^{-2}S/m$, respectively. The fluid field is square and the corresponding side length is set as L=20a. If the particle is neglected, the electric field is uniform, as the electric potential applied on the segment AB is 20 V, and CD is 0 V.

The finite-element mesh deforms while following the DEP motion of the particle in the ALE method, and its quality is reduced as the particle moves in the microfluidic channel. In this study, more than 20,000 total elements with a minimum of 100 elements positioned adjacent to the particle surface through rigorous mesh-refinement tests. In the simulation, the mesh element quality is a dimensionless quantity between 0 and 1, where 0 represents a degenerated element and 1 represents a perfectly regular element. The COMSOL (Stockholm, Sweden) computations include the default mesh quality measures method, which is used in this simulation. Before the mesh quality decreases below 0.7 out of the maximum 1.0 when the particle moves, the domain of the system is re-meshed with the current position of the particle. The computational process is restarted after the solution is mapped to the new mesh.

**Figure 2:**
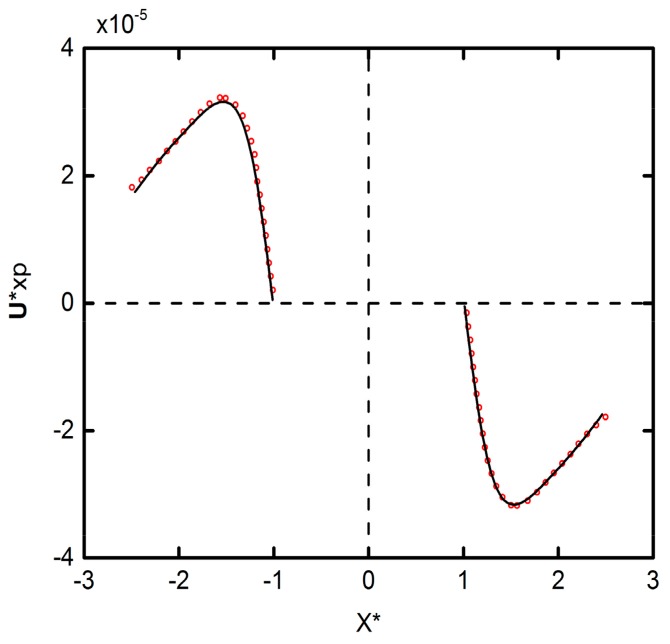
The velocity comparison of the two results. The lines and symbols represent results of this paper and Qian’s results [23], respectively.

In order to verify the accuracy of our simulation results, the horizontal DEP motion velocity graph are compared with Qian’s results [23], as shown in Figure 2. Note that the deformability of the particles is set to be the same as Qian’s, that is, as rigid particles. As can be seen from the figure, our simulation results are fully consistent with Qian’s results.

## 3. Results and Discussion

### 3.1. The Dielectrophoresis (DEP) Particle–Particle Interaction in the Horizontal Direction

**Figure 3:**
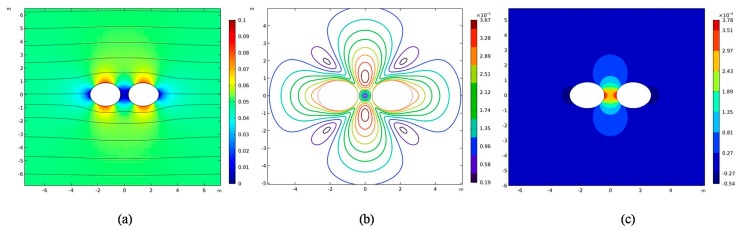
Distributions of the electric field (**a**), fluid field (**b**), and pressure (**c**) around two particles located at (x*,y*)=(±1.56,0) subjected to an external electric field E*=0.05. Lines in (**a**–**c**) represent, respectively, the streamlines of the electric field, flow field, and pressure.

The distribution of two particles in a fluid medium is usually random. Both horizontal (*θ* = 0°) and vertical positions (*θ* = 0°) are considered. The DEP interaction in the horizontal direction is studied in this section, and the vertical DEP interaction will be discussed in the next section. Figure 3a shows the distribution of electric fields in the entire computational domain, as can be seen, since the two particles exist simultaneously, causing the non-uniform electric field around a single particle. The non-uniform electric field polarizes the surface charge of the particles, so the corresponding DEP force is produced to drive the particle motion. Since a single particle does not cause a distortion of the electric field, it does not produce the corresponding DEP force, so we call the DEP force here the particle–particle interaction DEP force. In Figure 3a, DEP motion here can be called negative DEP motion from the higher electric field to the lower, causing the two particles to attract each other in the horizontal direction. At the same time, the DEP motion causes the distortion of fluid field, forming a symmetric circular flow field correspondingly, as shown in Figure 3b. With the distance between two particles decreasing, the fluid between the two particles is constantly compressed, leading to an increasing fluid pressure between the two particles, corresponding to the increasing hydrodynamic pressure force, as shown in Figure 3c.

**Figure 4:**
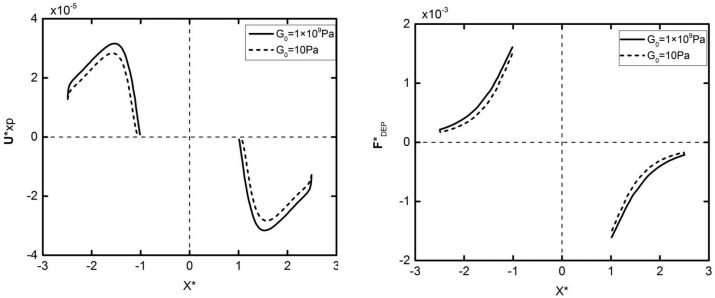
Velocity (**a**) and DEP particle–particle interaction force (**b**) variations of the two particles initially located at (x*,y*)=(±2.5,0) subjected to an external electric field E*=0.05. The solid lines and dashed lines in (**a**,**b**) represent *G*_0_ = 1 × 10^9^ Pa and *G*_0_ = 10 Pa, respectively.

**Figure 5:**
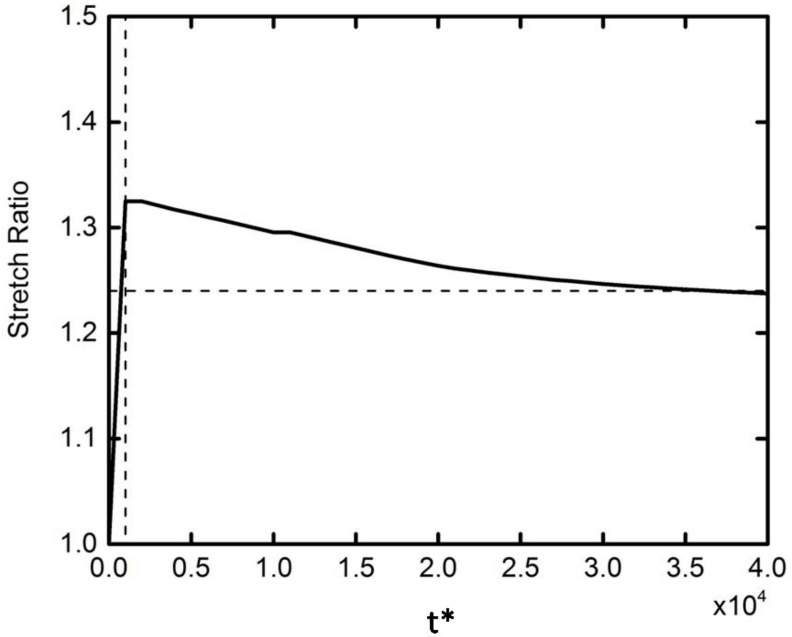
Stretch ratio *D* = *b*/*c* as a function of *D* = *b*/*c*, t*, where *b* and *c* represent the semimajor axis and the semiminor axis of the deformable ellipse, *G*_0_ = 10 Pa.

Figure 4 shows the velocity and DEP force in the horizontal direction of different elastic modulus particles with the same initial position. It can be seen from the figure that the DEP motion trends for different elastic modulus particles are the same, which can be divided into two stages. In the first stage, the DEP force increases as the particles approach each other (as shown in Figure 4b), leading to an increasing velocity. In the second stage, the hydrodynamic pressure force increases faster than the DEP force due to the further motion, leading to the decrease in the velocity to zero. Note that the DEP force of the deformed particles is smaller than the rigid particles, resulting in a smaller maximum speed. It can also be concluded that the two kinds of particles with different elastic modulus achieve a different assembly position when the velocity is zero, which is caused by the deformation of the particles. Figure 5 shows that the particle deformation process is rapidly completed in a very short time at the beginning of the DEP motion. The two spherical particles become ellipsoid, and their stretch ratio (the ratio of the long axis to the short axis) have a maximum value of about 1.32. The ratio is then reduced to around 1.24, and the two particles finally accomplish the whole DEP motion in a constant shape.

### 3.2. The DEP Particle–Particle Interaction in Vertical Direction

**Figure 6:**
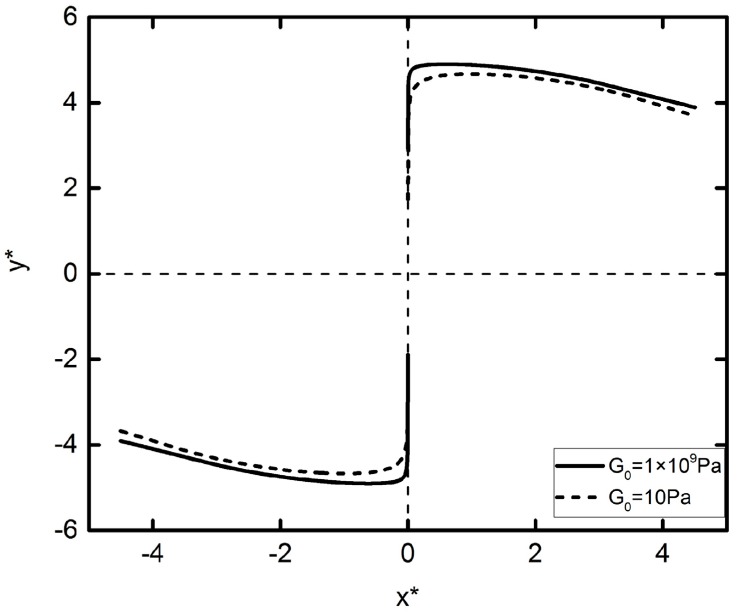
Trajectories of two particles initially located at (x*,y*)=(0,±1.5) subjected to an external electric field E*=0.05. The solid lines and dashed lines represent *G*_0_ = 1 × 10^9^ Pa and *G*_0_ = 10 Pa, respectively.

The DEP motion of the two particles perpendicular to the direction of the electric field is discussed. The initial position of the two particles is located at (x∗,y∗)=(0,±1.5), and the trajectory of their DEP motion is shown in Figure 6. The DEP motion in the vertical direction can also be divided into two stages. In the first stage, the two particles repel each other, causing the two particles to move in the opposite direction along the connection line. When the distance between two particles reaches a certain value, the DEP motion starts in the second stage, which the two particles start to attract each other, and rotate along the center point of their connection line. The turning points of the two stages are called “unstable points,” and the DEP motion direction of the two particles in this position is uncertain. Note that the DEP motion trends for different elastic modulus particles are the same, but the maximum movement distance of the deformation particles is lower.

**Figure 7:**
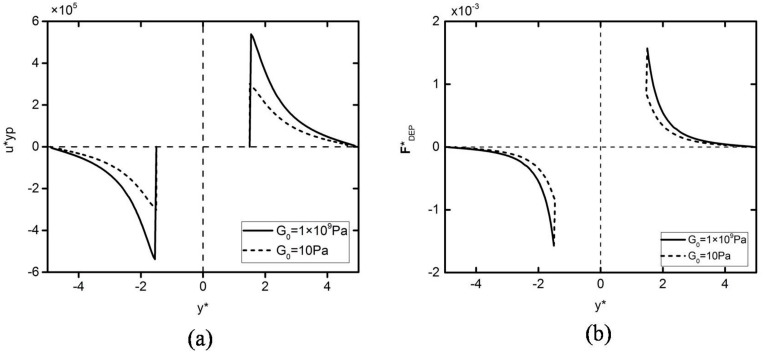
Velocity (**a**) and DEP particle–particle interaction force (**b**) variations of the two particles initially located at (x*,y*)=(0,±1.5) subjected to an external electric field E*=0.05. The solid lines and dashed lines in (**a**,**b**) represent *G*_0_ = 1 × 10^9^ Pa and *G*_0_ = 10 Pa, respectively.

The speed of DEP motion in the vertical direction is shown in Figure 7a. In a very short period of time, the particle is rapidly reaching the maximum speed from the stationary state, and the particle velocity then starts to decrease to zero. As can be seen, the maximum velocity of a deformed particle is smaller than that of a rigid particle. It can also be seen that the maximum velocity of a deformed particle DEP motion is smaller than that of a rigid particle. In order to explain the movement trajectory and velocity trend of different elastic modulus particles’ DEP motion, the DEP force graph in the vertical direction is shown in Figure 7b. As can be seen from the figure, the DEP force of the two particles is reduced to zero after reaching the maximum speed, but decreases faster at the beginning, and the velocity decreases firstly and then increases correspondingly. It can also be seen that the DEP force of the deformed particles is relatively smaller than the rigid particles. This is because the electric field distortion caused by the deformable particles is relatively mild, resulting in a relatively small polarization of the particles’ surface charge.

### 3.3. The DEP Particle–Particle Interaction in Arbitrary Direction

Considering the universality of the simulation results, the particle DEP interaction in any direction is studied. The DEP interaction motion in the direction of *θ* = 85° and *θ* = 45° will be discussed in the following sections. To further discuss, the elastic modulus, particle radius, and electric field intensity in this section will be simulated as a variable, and the results will be further discussed.

#### 3.3.1. DEP Particle–Particle Interaction: *θ* = 85° 

**Figure 8:**
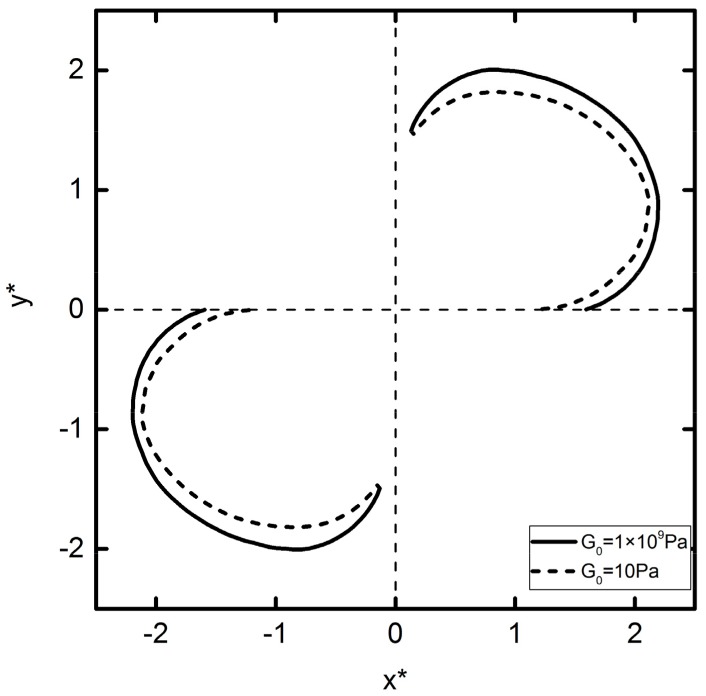
Trajectories of two particles initially located with *θ* = 85° and R* = 3 subjected to an external electric field E*=0.05. The solid lines and dashed lines represent *G*_0_ = 1 × 10^9^ Pa and *G*_0_ = 10 Pa, respectively.

The initial position of the two particles is located at (x*,y*)=(±1.5cos(85°),±1.5sin(85°)), and the DEP motion trajectory is shown in Figure 8. The DEP motion can be divided into two stages. In the first stage, the two particles repel each other, and the *y*-component DEP force repels the two particles to move in the opposite direction, and the *x*-component DEP attracts the two particles to rotate clockwise around the center point of their connection line. With the distance between two particles increasing, the two particles begin to attract each other and finally form a particle–particle chain parallel to the direction of the applied electric field, which is divided into the second stage of the DEP motion.

**Figure 9:**
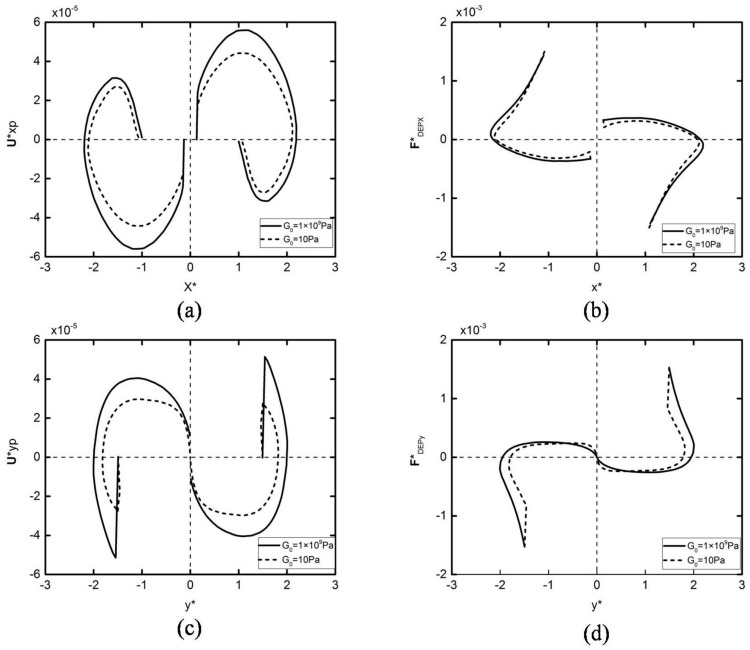
The *x*,*y*-component velocity (**a**,**c**) and *x*,*y*-component DEP particle–particle interaction force (**b**,**d**) variations of the two particles initially located with *θ* = 85° and R* = 3 subjected to an external electric field E*=0.05. The solid lines and dashed lines represent *G*_0_ = 1 × 10^9^ Pa and *G*_0_ = 10 Pa, respectively.

The DEP motion process described above can be interpreted by the DEP force graph, as shown in Figure 9. From Figure 9b,d, it can be concluded that DEP forces in the two stages have an opposite direction. If the first stage is assumed to be attractive force, then the second stage must be the repulsion force. Similarly, from Figure 9a,c, it can be concluded that *x* and *y* components of the DEP motion velocity undergo a process by which increase, decrease to zero, inverse increase, and decrease to zero. The particle velocity increases over a short period of time under the DEP repulsive force, and as the distance between the two particles increases further, the DEP force decreases to zero. The DEP force then turns into attractive force. Its *x*-component quantity attracts the two particles to continue rotating, and the *y*-component quantity attracts the two particles closer to each other. After the two particles are close to each other to a certain extent, the hydrodynamic pressure force between the particles increases, preventing the two particles from becoming any closer to each other, causing the particle velocity to decrease to zero. Finally, two particles form a particle–particle chain parallel to the electric field.

Note that different elastic modulus particles have different trajectories. The trajectories of the deformable particles are always inside the rigid particles. The larger the deformation is, the greater the degree of concavity becomes. The simulation results show that the finally assembly positions of deformable particles and rigid particles are different, and the deformable particles are closer together. Therefore, the simulation conclusion provides theoretical guidance for deformable particle DEP assembly.

#### 3.3.2. The Influence Factors of DEP Interaction: Particle Radius

**Figure 10:**
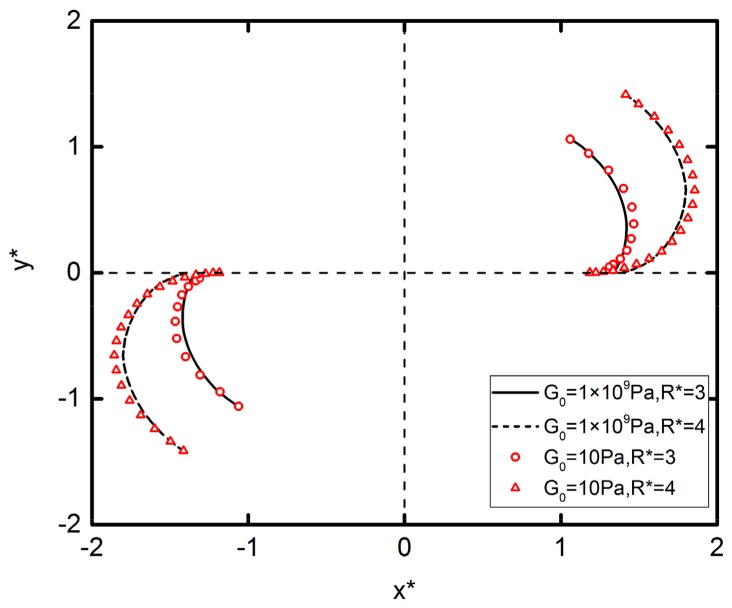
Trajectories of two particles initially located with *θ* = 45° and R* = 3 subjected to an external electric field E*=0.05. Lines and symbols represent *G*_0_ = 1 × 10^9^ Pa and *G*_0_ = 10 Pa, respectively. Solid lines and circles represent R* = 3. Dashed lines and triangles represent R* = 4.

The effect of particle radius on DEP interaction is briefly mentioned in the previous subsections, and this section provides concrete simulation results to further demonstrate the former conclusion, as shown in Figure 10. As can be seen, the greater the particle radius is, the longer the movement trajectory becomes. This is because the particles with a greater radius have a greater distortion of the electric field, which leads to a stronger surface charge polarization and a greater DEP force correspondingly. Different elastic modulus particles have different trajectories due to different DEP forces. Similarly, the effect of elastic modulus on DEP motion follows the former rules we discussed. Note that changing the particle radius can effectively perturb the DEP motion, and based on this, in the experiment of DEP particle assembly, the particles with different radius can achieve an ideal separation.

#### 3.3.3. The Influence Factors of DEP Interaction: Electric Field Intensity

**Figure 11:**
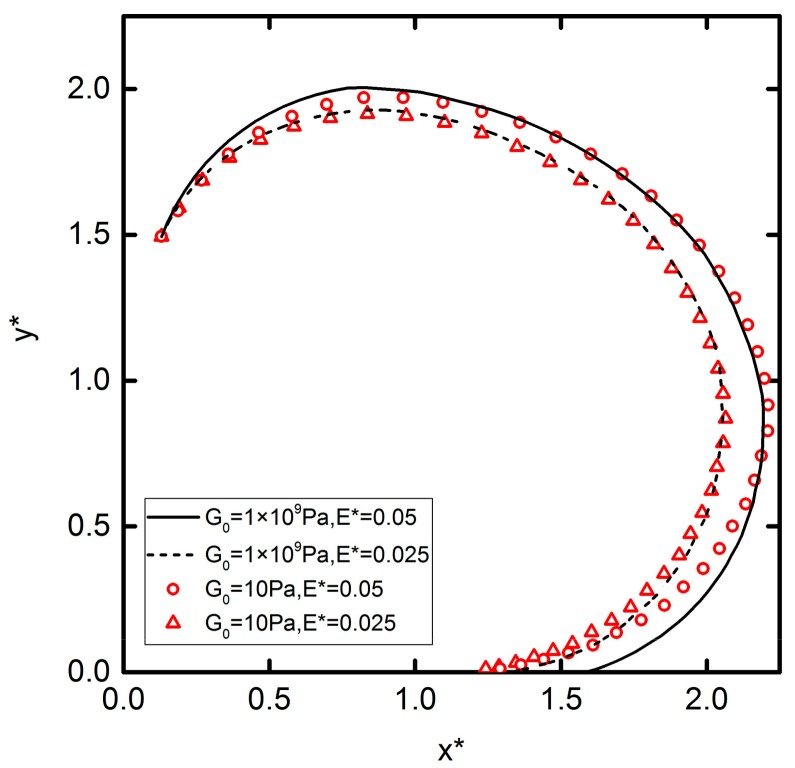
Trajectories of one particle initially located with *θ* = 85° and R* = 3. Lines and symbols represent *G*_0_ = 1 × 10^9^ Pa and *G*_0_ = 10 Pa, respectively. Solid lines and circles represent E*=0.05. Dashed lines and triangles represent E*=0.075.

Figure 11 shows the effect of electric field intensity on DEP motion. It can be concluded that the influence of elastic modulus on DEP motion is not obvious. On the contrary, the electric field intensity has an obvious disturbance effect on DEP motion, so changing the electric field can perturb the trajectory of the DEP motion effectively. The higher electric field intensity leads to a longer DEP motion trajectory. This is because a larger electric field intensity leads to a stronger polarization of the charge on the particles’ surface, causing greater DEP forces, that leads to more convex trajectories. Based on this phenomenon, in the actual experiment, the separation and assembly of different particles can be realized by setting different electric field intensity values.

## 4. Conclusions

Particle assembly based on DEP has become one of the main technologies of particle manipulation, and DEP particle interaction experiments and numerical simulations have been widely developed. In this paper, a mathematical model is developed based on the arbitrary Lagrangian-Eulerian (ALE) numerical approach method. The mathematical model can solve the electric field, the flow field, and the motion of deformable particles simultaneously. In the meantime, the MST method is used to calculate the DEP force by integrating the Maxwell stress tensor over the particle surface, which is regarded as the most accurate method to calculate DEP force. Simulation results show that, when the center distance is between two particles in a certain range, regardless of the initial position, the two deformable particles will eventually form a particle–particle chain parallel to the direction of the applied electric field. In the whole DEP motion, the DEP force changes with the distance between the two particles, and the hydrodynamic force accordingly changed. Based on this, the velocity of DEP motion is constantly changing. It can also be concluded that particle radius and electric field intensity have an important influence on DEP motion. Note that, compared with previous studies, the particles used in this study are deformable particles rather than rigid particles. The simulation results of this paper also show the differences between the two types of particle DEP motions. Because biological cells are deformable in actual experiments, the results of this simulation are more convincing. This paper therefore provides theoretical guidance for the DEP manual technology of biological cells, which can help to better understand the principle of DEP technology and promote the further development of DEP control technology. However, the simulation in the work only considers the hydrodynamic stress tensor and the Maxwell stress tensor. In order to model rather complex structures in future, some other forces or moments involved should be included in our model [34], especially if lubricants such as bovine serum albumin (BSA) are coating cells.

## References

[1] Zhu J., Tzeng T.-R.J., Hu G., Xuan X. (2009). DC dielectrophoretic focusing of particles in a serpentine microchannel. Microfluid. Nanofluid..

[2] Xuan X., Xu B., Li D. (2005). Accelerated particle electrophoretic motion and separation in converging-diverging microchannels. Anal. Chem..

[3] Ai Y., Park S., Zhu J., Xuan X., Beskok A., Qian S. (2010). DC electrokinetic particle transport in an L-shaped microchannel. Langmuir.

[4] Lu X., Hsu J.-P., Xuan X. (2014). Exploiting the wall-induced non-inertial lift in electrokinetic flow for a continuous particle separation by size. Langmuir.

[5] Xuan X., Zhu J., Church C. (2010). Particle focusing in microfluidic devices. Microfluid. Nanofluid..

[6] Xuan X., Raghibizadeh S., Li D. (2006). Wall effects on electrophoretic motion of spherical polystyrene particles in a rectangular poly (dimethylsiloxane) microchannel. J. Colloid Interface Sci..

[7] Ren Y., Liu W., Jia Y., Tao Y., Shao J., Ding Y., Jiang H. (2015). Induced-charge electroosmotic trapping of particles. Lab Chip.

[8] Yeh L.-H., Zhang M., Hu N., Joo S.W., Qian S., Hsu J.-P. (2012). Electrokinetic ion and fluid transport in nanopores functionalized by polyelectrolyte brushes. Nanoscale.

[9] Qian S., Bau H.H. (2005). Theoretical investigation of electro-osmotic flows and chaotic stirring in rectangular cavities. Appl. Math. Model..

[10] Ai Y., Mauroy B., Sharma A., Qian S. (2011). Electrokinetic motion of a deformable particle: Dielectrophoretic effect. Electrophoresis.

[11] Zhou T., Liu Z., Wu Y., Deng Y., Liu Y., Liu G. (2013). Hydrodynamic particle focusing design using fluid-particle interaction. Biomicrofluidics.

[12] Zhou T., Liu T., Deng Y., Chen L., Qian S., Liu Z. (2017). Design of microfluidic channel networks with specified output flow rates using the cfd-based optimization method. Microfluid. Nanofluid..

[13] Liang L., Ai Y., Zhu J., Qian S., Xuan X. (2010). Wall-induced lateral migration in particle electrophoresis through a rectangular microchannel. J. Colloid Interface Sci..

[14] Ai Y., Qian S., Liu S., Joo S.W. (2010). Dielectrophoretic choking phenomenon in a converging-diverging microchannel. Biomicrofluidics.

[15] Dash S., Mohanty S. (2014). Dielectrophoretic separation of micron and submicron particles: A review. Electrophoresis.

[16] Jubery T.Z., Srivastava S.K., Dutta P. (2014). Dielectrophoretic separation of bioparticles in microdevices: A review. Electrophoresis.

[17] Zhou T., Xu Y., Liu Z., Joo S.W. (2015). An enhanced one-layer passive microfluidic mixer with an optimized lateral structure with the dean effect. J. Fluids Eng..

[18] Zhou T., Ge J., Shi L., Fan J., Liu Z., Woo Joo S. (2018). Dielectrophoretic choking phenomenon of a deformable particle in a converging-diverging microchannel. Electrophoresis.

[19] Kale A., Lu X., Patel S., Xuan X. (2014). Continuous-flow dielectrophoretic trapping and patterning of colloidal particles in a ratchet microchannel. J. Micromech. Microeng..

[20] Hossan M.R., Dillon R., Roy A.K., Dutta P. (2013). Modeling and simulation of dielectrophoretic particle–particle interactions and assembly. J. Colloid Interface Sci..

[21] Xie C., Chen B., Liu L., Chen H., Wu J. (2016). Iterative dipole moment method for the interaction of multiple dielectrophoretic particles in an AC electrical field. Eur. J. Mech. B/Fluids.

[22] Yan Y., Morris J.F., Koplik J. (2007). Hydrodynamic interaction of two particles in confined linear shear flow at finite reynolds number. Phys. Fluids.

[23] Ai Y., Qian S. (2010). DC dielectrophoretic particle–particle interactions and their relative motions. J. Colloid Interface Sci..

[24] Ai Y., Zeng Z., Qian S. (2014). Direct numerical simulation of AC dielectrophoretic particle–particle interactive motions. J. Colloid Interface Sci..

[25] Kang K.H., Li D. (2006). Dielectric force and relative motion between two spherical particles in electrophoresis. Langmuir.

[26] Hwang H., Kim J.-J., Park J.-K. (2008). Experimental investigation of electrostatic particle−particle interactions in optoelectronic tweezers. J. Phys. Chem. B.

[27] Khoshmanesh K., Akagi J., Nahavandi S., Skommer J., Baratchi S., Cooper J.M., Kalantar-Zadeh K., Williams D.E., Wlodkowic D. (2011). Dynamic analysis of drug-induced cytotoxicity using chip-based dielectrophoretic cell immobilization technology. Anal. Chem..

[28] Zhou T., Yeh L.-H., Li F.-C., Mauroy B., Joo S. (2016). Deformability-based electrokinetic particle separation. Micromachines.

[29] Krueger T., Holmes D., Coveney P. (2014). Deformability-based red blood cell separation in deterministic lateral displacement devices-a simulation study. Biomicrofluidics.

[30] Korin N., Bransky A., Dinnar U. (2007). Theoretical model and experimental study of red blood cell (RBC) deformation in microchannels. J. Biomech..

[31] Beech J.P., Holm S.H., Adolfsson K., Tegenfeldt J.O. (2012). Sorting cells by size, shape and deformability. Lab Chip.

[32] Zhou T., Shi L., Fan C., Liang D., Weng S., Joo S.W. (2017). A novel scalable microfluidic load sensor based on electrokinetic phenomena. Microfluid. Nanofluid..

[33] Zhou T., Deng Y., Zhao H., Zhang X., Shi L., Woo Joo S. (2018). The mechanism of size-based particle separation by dielectrophoresis in the viscoelastic flows. J. Fluids Eng..

[34] Soffe R., Tang S.-Y., Baratchi S., Nahavandi S., Nasabi M., Cooper J.M., Mitchell A., Khoshmanesh K. (2015). Controlled rotation and vibration of patterned cell clusters using dielectrophoresis. Anal. Chem..

